# Delays in the post-marketing withdrawal of drugs to which deaths have been attributed: a systematic investigation and analysis

**DOI:** 10.1186/s12916-014-0262-7

**Published:** 2015-02-05

**Authors:** Igho J Onakpoya, Carl J Heneghan, Jeffrey K Aronson

**Affiliations:** Nuffield Department of Primary Care Health Sciences, Centre for Evidence-based Medicine, University of Oxford, New Radcliffe House, Radcliffe Observatory Quarter, Oxford, OX2 6GG UK

**Keywords:** Death, Drug withdrawal, Review, Voluntary recall

## Abstract

**Background:**

Post-marketing withdrawal of medicinal products because of deaths can be occasioned by evidence obtained from case reports, observational studies, randomized trials, or systematic reviews. There have been no studies of the pattern of withdrawals of medicinal products to which deaths have been specifically attributed and the evidence that affects such decisions. Our objectives were to identify medicinal products that were withdrawn after marketing in association with deaths, to search for the evidence on which withdrawal decisions were based, and to analyse the delays involved and the worldwide patterns of withdrawal.

**Methods:**

We searched the World Health Organization’s Consolidated List of [Medicinal] Products, drug regulatory authorities’ websites, PubMed, Google Scholar, and textbooks on adverse drug reactions. We included medicinal products for which death was specifically mentioned as a reason for withdrawal from the market. Non-human medicines, herbal products, and non-prescription medicines were excluded. One reviewer extracted the data and a second reviewer verified them independently.

**Results:**

We found 95 drugs for which death was documented as a reason for withdrawal between 1950 and 2013. All were withdrawn in at least one country, but at least 16 remained on the market in some countries. Withdrawals were more common in European countries; few were recorded in Africa (5.3%). The more recent the launch date, the sooner deaths were reported. However, in 47% of cases more than 2 years elapsed between the first report of a death and withdrawal of the drug, and the interval between the first report of a death attributed to a medicinal product and eventual withdrawal of the product has not improved over the last 60 years.

**Conclusions:**

These results suggest that some deaths associated with these products could have been avoided. Manufacturers and regulatory authorities should expedite investigations when deaths are reported as suspected adverse drug reactions and consider early suspensions. Increased transparency in the publication of clinical trials data and improved international co-ordination could shorten the delays in withdrawing dangerous medicinal products after reports of deaths and obviate discrepancies in drug withdrawals in different countries.

Please see related article: http://dx.doi.org/10.1186/s12916-015-0270-2.

**Electronic supplementary material:**

The online version of this article (doi:10.1186/s12916-014-0262-7) contains supplementary material, which is available to authorized users.

## Background

When a medicinal product is found to cause death as an adverse reaction, one would expect expedited investigation of the problem and, when indicated, withdrawal of the product from the market. However, the relation between occurrences of deaths and the time to withdrawal of responsible products has not hitherto been studied.

When drug regulatory authorities consider that a medicinal product has a favourable benefit-to-harm balance, they award a licence and the product becomes available [1]. However, some adverse reactions are discovered only after approval and marketing [2,3], in which case regulators have several possible courses of action, depending on the risk and seriousness of the adverse reaction. They can:

- Require the reaction to be added to the label (§4.8, “Undesirable effects”, in the EU Summaries of Product Characteristics) [4];
- Require the addition of a warning (§4.4, “Special warnings and precautions for use”) [4];
- Require the addition of a contraindication (§4.3), if applicable [4];
- Allow the patient, informed by the prescriber, to decide whether to use the drug;
- Require an amendment to any Post-Authorization Safety Study that is being performed as a condition of the licence;
- Require a revision of specific risk minimization measures mentioned in the product’s Risk Management Plan;
- Require the marketing authorization holder (MAH) to issue a Direct Health-care Professional Communication (e.g., a “Dear John” letter);
- In the USA, require the use of a Black Box warning, which confirms that the drug carries a significant risk of a serious adverse reaction.

The MAH may take some of these actions themselves without being required to do so by the regulators. The final regulatory action would be to suspend or revoke the licence, and the MAH sometimes withdraws a drug voluntarily before being forced to by the regulator. These options are not mutually exclusive and can be undertaken sequentially or in parallel, depending on the case and urgency.

Post-marketing withdrawal of a medicinal product because of drug-attributed deaths can be occasioned by evidence obtained from individual case reports or case series, observational studies, randomized comparisons, or systematic reviews. Withdrawal of products from the market because of deaths can sometimes be controversial, especially when a causal connection between drug use and deaths has not been clearly established. It can also lead to substantial financial losses for manufacturers, giving negative incentives.

There have been no studies of the pattern of withdrawals of medicinal products to which deaths have been specifically attributed and the evidence that affects decisions. Building on a preliminary analysis of 284 medications that have been withdrawn or had major changes to their labels following reports of adverse drug reactions, including deaths [5], we have identified medicinal products that have been withdrawn in the last 60 years in association with deaths, have searched for the evidence on which the withdrawal decisions were based, and have analysed the delays and the worldwide patterns of (drugs) withdrawal.

We use the terms “withdrawal” and “withdrawn” to indicate that the product has either been voluntarily withdrawn by the MAH or had its licence revoked by a regulatory agency. Other terms that are sometimes used to describe the latter include “banned”, “prohibited”, “recalled”, and “not (i.e., no longer) approved”.

## Methods

We identified products for which death was documented as a reason for withdrawal between 1950 and December 2013, from the following sources:

- The World Health Organization’s (WHO’s) database of Consolidated List of Products whose consumption and/or sale have been banned, withdrawn, severely restricted, or not approved by governments (Issues 12 and 14);
- The WHO’s *Drug Information* (Volumes 1–27);
- The WHO’s *Pharmaceuticals Newsletter* (1997–2013);
- The database of withdrawn drugs of the European Medicines Agency;
- The website of the UK Medicines and Healthcare products Regulatory Agency (MHRA);
- The website of the US Food and Drug Administration (FDA);
- PubMed, MEDLINE, and Google Scholar, using search terms including “drug withdrawal”, “fatal*”, “death(s)”, “voluntary recall”, and related terms;
- *Meyler’s Side Effects of Drugs* volumes 1–8 and editions 9–15 and the *Side Effects of Drugs Annuals* 1–35;
- *Stephens’ Detection of New Adverse Drug Reactions*, 5th edition [6].

A Medline search strategy is available as Additional file 1.

To be included in the review, a product must have had death documented as a reason for withdrawal, irrespective of whether the deaths had occurred at therapeutic or toxic doses. Medicines that had previously been withdrawn in association with deaths but had been re-introduced or made available in other, safer, formulations were included. Products for which death was not specifically mentioned as a reason for withdrawal from the market were excluded, as were non-human medicines, herbal products, and non-prescription medicines.

For each withdrawn product, we extracted data on the date of marketing authorization, launch date, or date of first recorded use; the drug class or therapeutic indication [7]; the date of the first reported death; the first withdrawal date; the country or countries of withdrawal, even if death was not reported as a reason for withdrawal in all the countries; and the reported mechanism by which the drug resulted in death. We took the view that if death was reported as a reason for withdrawal in at least one country, this was sufficient for inclusion of other countries in which the drug had been withdrawn since it was likely to have caused deaths elsewhere, even if not cited as such. When the exact launch date of a drug was not available (two cases), its first record of use in humans, based on a PubMed search, was used as its launch date.

We used the WHO Consolidated List as the primary source of information for dates of first launch and first withdrawal (if reported). These dates were cross-checked with the corresponding dates from the other sources listed above.

We documented the level of available evidence for reports of deaths related to the withdrawn drug using the tool kit of the Oxford Centre for Evidence-based Medicine [8]. One reviewer [IJO] extracted the data and a second reviewer [JKA] verified them independently. When there were discrepancies in the attributed dates (three instances), the reviewers re-checked the dates together and arrived at a consensus through discussion.

Scatter plots were used to explore the relationship between launch years and delays to first reports of death or first withdrawal. Pearson’s χ^2^ tests were used to compare differences in the frequencies of withdrawals within 1 year of the report of death; *P* values <0.05 were considered significant.

## Results

**Figure 1:**
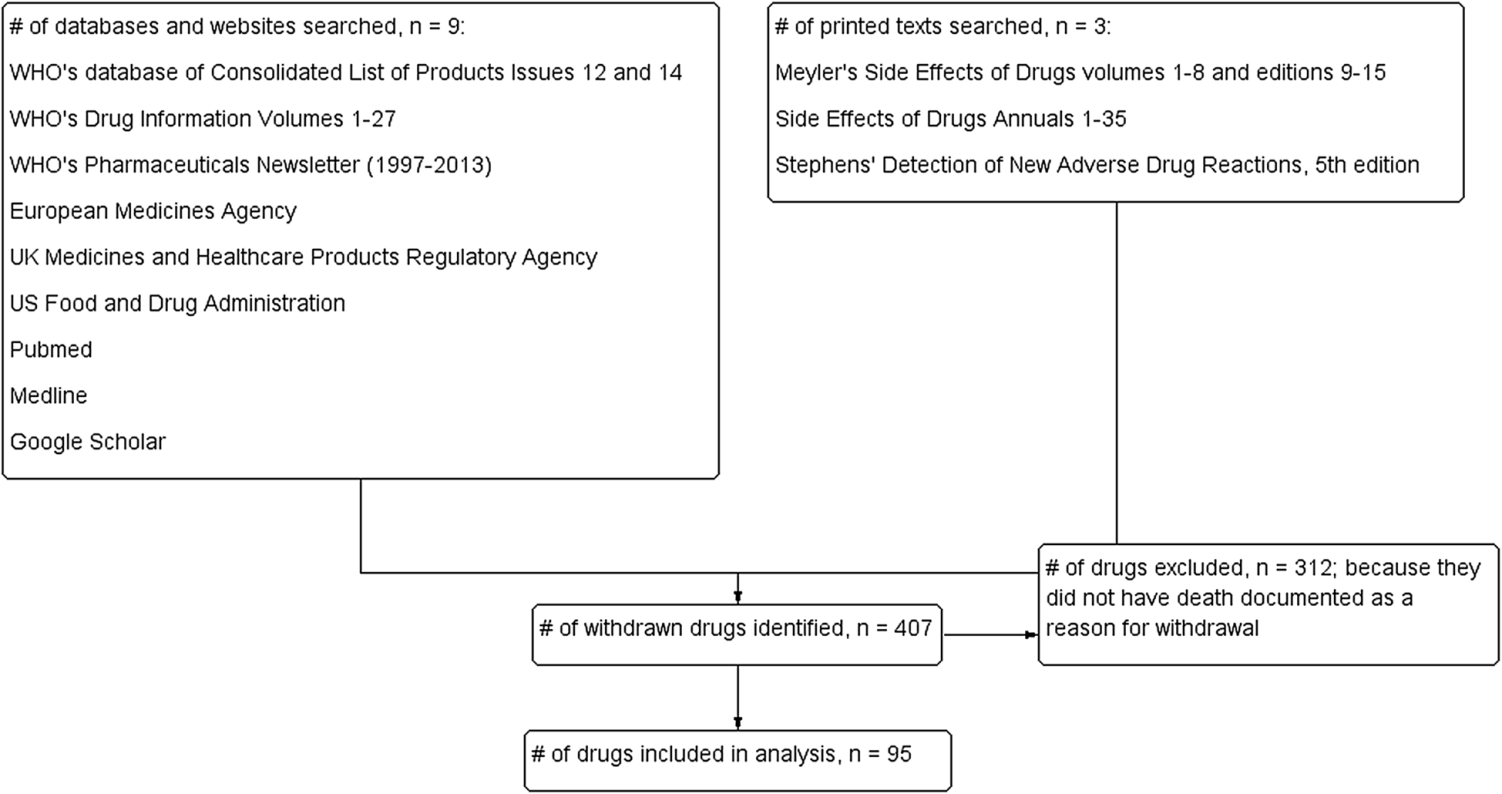
**Schematic diagram showing process for inclusion of medicinal products withdrawn after approval because of drug-attributed deaths.**

We identified 407 medicinal products withdrawn worldwide (Figure 1). Out of these, we excluded 312 products because death was not reported as a reason for withdrawal. Finally, we included 95 products for which death was documented as a reason for withdrawal in the review (Additional file 2). Most of the drugs were used to treat neurological or psychiatric disorders (n = 28) or as analgesics or anti-inflammatory drugs (n = 21); 14 were used for cardiorespiratory disorders and 9 were antimicrobial drugs.

The withdrawals occurred between 1957 and 2011. The longest interval between launch year or year of first reported use and the first report of fatality was 127 years (ethyl nitrite; first reported use 1850, first reported death 1977) [9], and the longest interval between the first reported death and withdrawal of the medicine was 56 years (phenobarbital; first reported death 1929, withdrawn 1985 in Sweden [10]).

At least 40% of the drugs were withdrawn in more than one country, but only 26 (27%) were reportedly withdrawn “worldwide” (i.e., in all the countries in which they had been marketed). Sixteen products continued to be marketed in some countries, despite having been withdrawn in at least two others (Additional file 2). Twelve products (13%) were withdrawn because they caused deaths after overdose.

**Figure 2:**
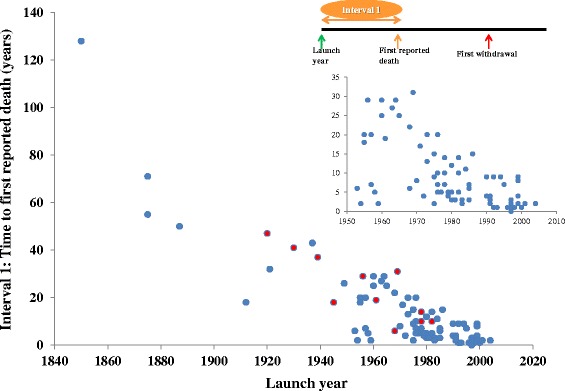
**Launch year versus interval 1 (the time lapse between the launch of a withdrawn product and the date of the first reported death).** The red circles indicate medicinal products that were withdrawn due to overdose-related deaths.

**Figure 3:**
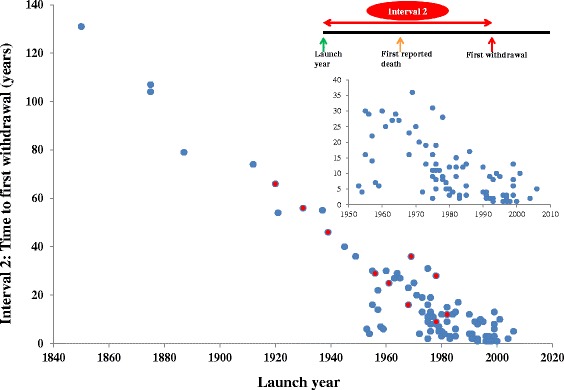
**Launch year versus interval 2 (the time lapse between the launch of a withdrawn product and the date of the first reported withdrawal).** The red circles indicate medicinal products that were withdrawn due to overdose-related deaths.

Figure 2 shows the time lapse between the launch of a withdrawn product and the date of the first reported death (interval 1); the more recent the launch date, the sooner deaths were reported. Figure 3 shows the time lapse between the launch of a withdrawn product and the date of the first reported withdrawal (interval 2), which has also shortened over time. In both cases, the finding was similar when the 12 products withdrawn because of deaths due to overdose were examined. These findings applied to the data after 1950 as well as to the whole set shown (see insets in Figures 2 and 3). In cases where deaths were due to overdose, the average time to withdrawal after the first report of death was 13 years; the mean withdrawal interval when deaths occurred at therapeutic doses was 4 years. We observed similar results when the interval between launch year and first withdrawal year were examined (35 years vs. 15 years).

**Figure 4:**
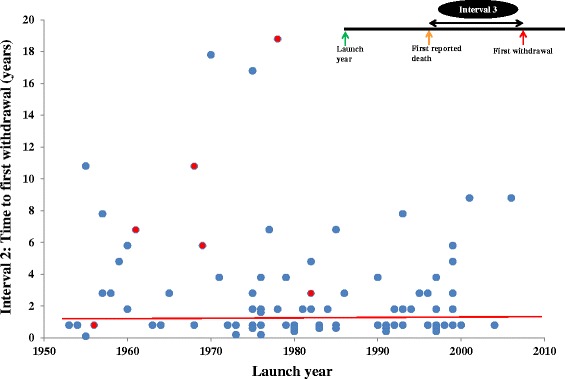
**Launch year versus interval 3 (the time lapse between the first reported death and the first withdrawal in any country).** The red circles indicate medicinal products that were withdrawn due to overdose-related deaths.

However, the delay between the first death and the first withdrawal (interval 3) showed no consistent relation to the year in which the product was launched, and did not change consistently over time (Figure 4). Of 81 products launched after 1950, 31 (38%) were withdrawn within 1 year of the first reported death, 12 (15%) within the second year, and 38 (47%) more than 2 years after the first reported death. Similarly, the delay between the first death and the first withdrawal showed no consistent relation to the year in which the first death was reported when six medicinal products withdrawn after 1950 because of deaths due to overdose were examined (Figure 4).

There were significantly fewer withdrawals in African and Asian countries than in other countries when comparing medications that were withdrawn within 1 year of the first report of a death and those that were withdrawn later (*P* <0.0005). The frequency of respiratory events that occasioned withdrawal was also significantly lower in the former (*P* = 0.04).

The evidence for withdrawal in most cases came from case reports (79/95; 83%). In three cases, the evidence was based on the results of case-control studies and in eight cases on randomized controlled comparisons. In one of those cases (rosiglitazone) there was also evidence from cohort studies and a systematic review. The evidence for withdrawal of two drugs (celecoxib and rofecoxib) was based on the results of randomized comparisons or meta-analysis.

Of published reports of deaths, 72 (76%) came from physicians; in 17 cases (18%) deaths were reported to regulatory agencies, and in two cases in clinical trials; other sources included a government inquiry and a coroner’s report.

Cardiotoxicity (n = 18), hepatotoxicity (n = 25), and respiratory depression (n = 10, all of which were attributed to overdose with central nervous system depressants), accounted for 56% of withdrawals. Two drugs (mibefradil and sorivudine) were withdrawn because interactions with other medications resulted in deaths. Three drugs (bicalutamide 150 mg, fenoterol, and nebacumab) were withdrawn because “accelerated deaths” were reportedly associated with their use, while pumactant and flosequinan were withdrawn because of increased mortality.

Boric acid was withdrawn because of deaths from systemic absorption associated with its use as a topical antiseptic. Pituitary-derived somatropin was withdrawn because of deaths due to Creutzfeldt-Jakob disease. L-tryptophan was withdrawn because of the eosinophilia-myalgia syndrome with resultant deaths.

## Discussion

We have identified 95 products for which death was cited as a reason for withdrawal between 1950 and 2013. Cardiotoxicity, hepatotoxicity, and respiratory depression accounted for over half of the withdrawals. Withdrawals were more common in European countries; few were reported in Africa.

### Evidence for withdrawal

The criteria that determine whether a product should be withdrawn are not well established, although proposals have been made [11]. It is likely that in many cases agencies have applied the precautionary principle, defined by the Wingspread Consensus Statement [12], as follows: “*When an activity raises threats of harm to human health … precautionary measures should be taken, even if some cause and effect relationships are not fully established scientifically,* [in which case] *the proponent of* [the] *activity … should bear the burden of proof*”. The “proponent” in this case would be the MAH.

The quality of the evidence that led to the withdrawal of these medicinal products was on the whole poor. In most instances, it was limited to case reports, and in only eight instances was it based on randomized comparisons. This is consistent with the results of much smaller previous studies. Of 11 products that were withdrawn for different reasons during 1999 to 2001, evidence from spontaneous reports supported the withdrawal of eight products (73%); randomized trials and comparative observational studies were cited for only two products each [13]. In a study of 19 products that were withdrawn during 2002 to 2011 (at least two studies were reported as evidence used for withdrawal of 10 products), case reports were cited in 18 of 19 withdrawals, case-control studies in four cases, cohort studies in four, randomized controlled trials in 12, and meta-analysis in five [14]. Similarly, in a Spanish study of 22 drugs that were withdrawn during 1990 to 1999 because of adverse reactions, case reports were the main source of information [15].

In the world literature, about 30% of all information on adverse drug reactions comes from case reports [16], but in our survey 83% of withdrawals were based on such evidence. This is consistent with the observation that suspected adverse reactions that are identified in case reports are infrequently followed-up with formal studies to investigate the suspected associations; of 63 suspected adverse drug reactions that were the subjects of case reports in five medical journals in 1997, 52 were not subjected to further detailed evaluation and data from controlled studies that supported the postulated link between the product and the adverse event were available in only three cases [17]. However, when death is suspected to result from the use of a medicine, one might apply less strict criteria in attributing causality than for other, less serious, adverse reactions, in order to give higher priority to their investigation.

Nevertheless, for some of the medicines in this study, a cause and effect relation could be established. For example, overdose with sedative drugs (e.g., pentobarbital and co-proxamol, with consequent respiratory paralysis and death) led to withdrawals [18]. For some other drugs, such as fenoterol and nebacumab, a cause and effect relation was established indirectly by analysing patterns of mortality in patients taking the drugs. In some cases, retrospective evidence of an association was also available; for example, a marked reduction in mortality rates from asthma was reported after fenoterol was removed from the market in New Zealand [19]; after L-tryptophan was withdrawn in 1989, when epidemiological studies linked its use to eosinophilia-myalgia syndrome, the incidence fell markedly [20]. In other cases [21,22], the cause and effect relation was not clearly demonstrated, and there have been controversies about whether such agents should have been removed from the market.

Negative publication bias is possible when deaths result from the use of medicinal products. For example, when rofecoxib was withdrawn, the manufacturer’s statement referred to unpublished trial data and did not mention deaths [23]. While regulatory authorities in Europe withdrew nefazodone because of hepatotoxicity, the manufacturer stated that they withdrew the product for commercial reasons [24]. In the case of dithiazanine iodide, only two of eight deaths resulting from the use of the product were published; the other six were noted in a pharmaceutical company’s brochure [25]. More recently, a drug manufacturer received a large fine for withholding data on the safety of a medicinal product reported to have caused numerous deaths [26]. Another manufacturer is also under investigation for the non-reporting of deaths from patient support programmes [27].

### Patterns of withdrawal

There were differences in the patterns of withdrawal in different countries. For example, in the UK troglitazone was withdrawn because of deaths due to liver damage, but in the USA the label was changed to require more extensive monitoring of liver function. Furthermore, there were significantly fewer withdrawals in African and Asian countries than in other countries, when comparing medications that were withdrawn within 1 year of the first report of a death and those that were withdrawn later.

**Figure 5:**
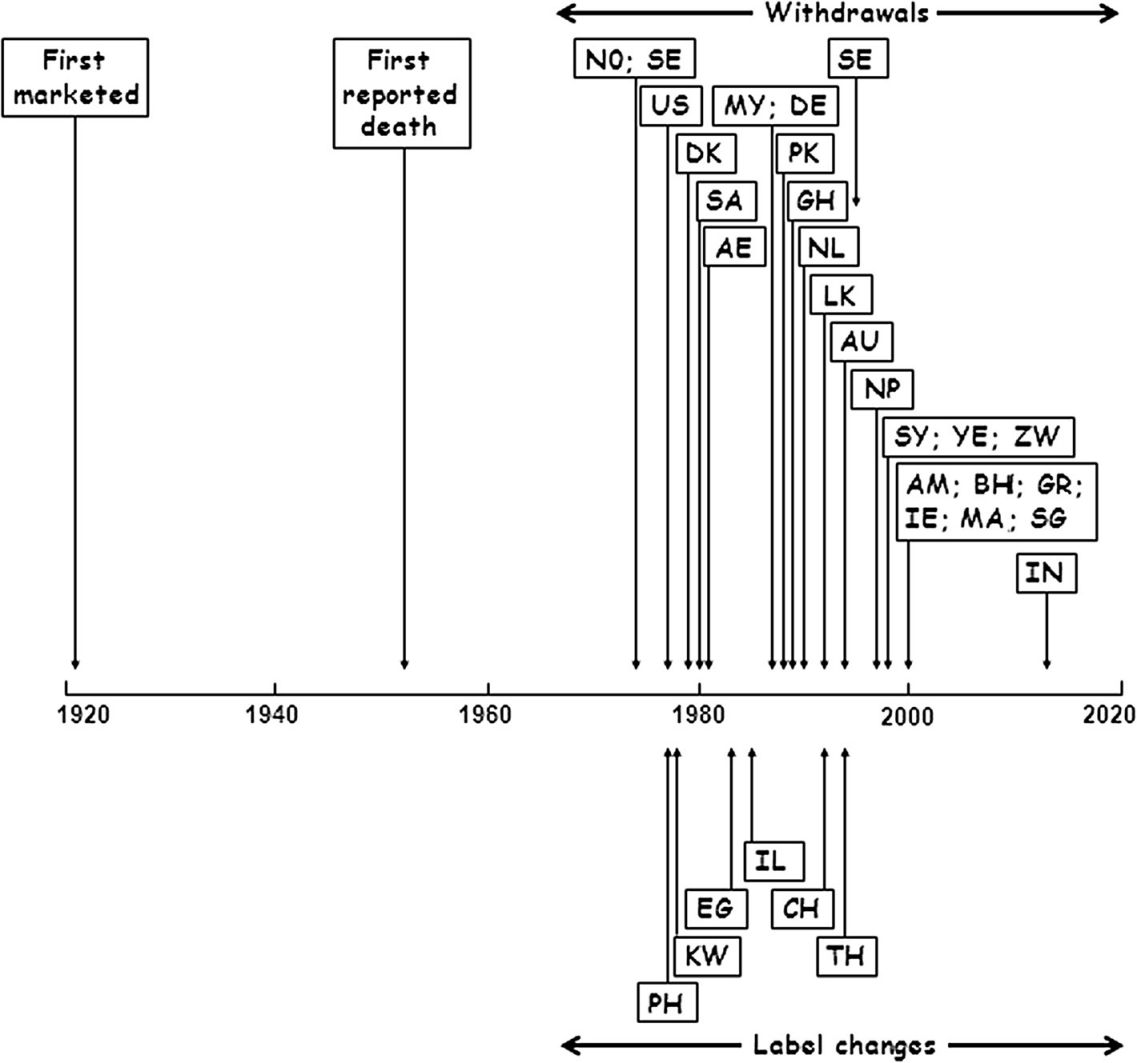
**Different times of withdrawal of metamizole in different countries.**

Only 26 products were reportedly withdrawn “worldwide”, i.e., in all countries in which they had been marketed, although it is unlikely that they were marketed everywhere. In 16 cases that we could identify, the product continued to be marketed in some countries, despite having been withdrawn in others. Metamizole (dipyrone), which has been the subject of considerable controversy [28], provides an excellent example of this (Figure 5). Although the first death was recorded in 1952, metamizole was first withdrawn from the market (in Norway and Sweden) only in 1974, and other countries were slower to withdraw it. Indeed, it was reintroduced in Sweden in 1995 and withdrawn again in 1999, and it has only recently been withdrawn in India [29]. In other countries, it remained on the market with changes to the label. In some cases, combination formulations were withdrawn but single-product formulations remained.

Several reasons explain the withdrawal of a product in one country and not in another. The frequency of an adverse reaction may differ in different countries, as was controversially reported with metamizole [30]. In a few cases different indications for therapy may contribute to different risks of adverse reactions. In other cases, the product may be cheaper than alternatives; there is a relation between a country’s capacity to restrict dangerous drugs and its per caput gross national product [31]. Furthermore, competing interests may play a role in influencing the pattern of drug withdrawal, even when deaths have been associated [32]. A recent analysis of five of the largest private global health foundations concluded that board members’ interests and donors’ investments (including those from pharmaceutical companies) are potential conflicts of interest that need to be addressed in order to prevent a distortion of science and public health outcomes [33]. Whether an independent group of reviewers should be tasked with making decisions about drug withdrawals because of adverse reactions is controversial. In the USA, for example, some authors have advocated that an independent group of reviewers should be mandated with critically appraising and analysing post-marketing surveillance data [32,34]; this view has been contradicted by others who prefer that the FDA be bolstered in its drug monitoring strategies [35].

### Delays between launch dates and reports of deaths

Several factors may have contributed to shortening the delay between the development and marketing of a drug and the first report of a drug-attributed death (interval 1). These include the likelihood that more patients are being treated nowadays with each new drug than was the case in the past, the increasing age of the treated population, and the increasing use of polypharmacy, whether appropriate or inappropriate. Improved pharmacovigilance and better reporting of suspected adverse reactions, better methods of signal detection and assigning causality, and stricter regulation could also contribute.

#### Improved pharmacovigilance

Modern pharmacovigilance dates from the 1960s [36,37], and developments over the last 50 years include the acquisition of large numbers of case reports in international databases, combined with increasing use of statistical methods to analyse agglomerated case reports, endowing them with greater evidential power [38], the increasing use of meta-analysis of adverse drug reaction reports in clinical trials [39], and the introduction of patient reporting.

#### Causality assignment

Some adverse drug reactions are definitive (“between-the-eyes” reactions) [40] and some are so-called “designated medical events” (i.e., reactions that are almost always or very often associated with a medicine when they occur) [41]. In this series, a death that occurred within minutes of injection of technetium (^99m^Tc) fanolesomab [42] and a death that occurred immediately after the intravenous administration of bismuth tartrate [43] fell into the first of these categories. For other reactions, several methods of determining causality (i.e., linking drug-adverse event pairs causally) have been devised over the last 35 years or so [44]. However, there are many drawbacks to their use [45]: they all rely to some extent on clinical judgement, and physicians tend to overestimate the likelihood of causality; the criteria used are neither sensitive nor specific and have poor predictive power; and in comparisons of different algorithms, agreement has generally been very poor. It therefore seems unlikely that improvements in causality assessment in individual cases have contributed much to the improved time to withdrawal of products that cause deaths.

### Delays between launch dates and withdrawals

The delay between launch date and market withdrawal (interval 2) shortened as the launch date became more recent (Figure 3). This is probably attributable to the shortening in the delay between launch and the first report of death (i.e., interval 1; Figure 2). One would expect faster withdrawal of a drug from the market if it caused deaths at therapeutic doses compared with another product that caused deaths through overdose, and the evidence from our review supports this assumption – the average times to withdrawal from both launch dates and first reports of deaths were longer with medicinal products that caused deaths through overdose.

### Delays to withdrawals after reports of deaths

In contrast, there was no consistent change in the delay between reports of death and market withdrawals (interval 3; Figure 4). Manufacturers and regulatory agencies need to address this problem, by putting in place processes to ensure rapid and internationally coordinated responses about decisions regarding withdrawal of medicinal products from the market if deaths are suspected to be associated with their use. However, it should be noted that the market share and profitability of particular drugs would influence the industry’s attitude to withdrawal.

Drug regulatory activity continues to evolve [46]. For example, it is a current international regulatory requirement that fatal or life-threatening unexpected adverse reactions must be reported within 15 days [47]. However, there are discrepancies in the ways in which individual regulatory authorities expedite action on such reports. In Europe, the USA, India, and Australia there are established standard procedures for initiating drug withdrawals from the market because of deaths [48-51]. For instance in the USA, if there is a reasonable risk of death or other serious adverse reactions from use of a medicinal product, the FDA may ask the MAH to recall a drug, or mandate the MAH to conduct a recall (Class I recall), and may also issue a safety alert to notify the general public about the hazards of the drug [49].

In a 1984 review of 24 products that were withdrawn during 1974 to 1983, there was no difference in the rates of withdrawals in the UK and the USA [52]. However, in an investigation of 26 medicinal products that were withdrawn in the USA and/or UK during 1971 to 1992, products that were withdrawn for any reason in the USA were withdrawn sooner after marketing than in the UK, which was attributed to more stringent regulation in the USA, with slower regulatory approval [53]. However, our data suggest that, at least as far as products that cause deaths are concerned, changes in product licensing in recent years have not shortened the times to withdrawal. This might, however, be in part due to increased delays in the regulatory process, occasioned by waiting for the results of further studies into the nature of the suspected adverse reaction. This implies that while such studies are being carried out, the medication should be temporarily withheld pending the results (see recommendations below).

### Limitations

We have no information on the time lapse between the occurrence of the first death attributed to the drug and the date on which death was first reported. Indeed, some early deaths may have been observed but not reported. However, such delays are likely to have been short and unlikely to have affected the results significantly.

Our list of 95 drugs withdrawn may be incomplete because of negative publication bias. We have identified about 300 other products that were also withdrawn or had their labels changed during the period we studied; some of those may have caused deaths, but since death was not specifically mentioned in relation to withdrawals in those cases, we have excluded them.

We do not have data on regulatory actions that may have been taken in advance of withdrawal. Some of the withdrawn drugs included in the review may also have been marketed as over the counter medications in some countries, and in countries in which regulation is less stringent, regulatory action would be more difficult. Furthermore, some medicinal products (especially psychotropic drugs) are reported to have caused deaths, but have not been withdrawn from the market. This may have been because the benefit-to-harm balance was nevertheless still considered to be favourable or because of poor regulatory action.

Our conclusions about international patterns of drug withdrawals are limited by the relative paucity of data in some countries.

### Recommendations

These data suggest that better methods are needed to detect, document, and report deaths in patients taking medications in order to reduce delays further, and especially for deciding how to deal with a product after deaths have been reported. We advocate a more robust approach to decision making regarding reports of deaths, and increased collaboration and co-ordination between agencies. Increased transparency in the publication of clinical trials data would help [54].

Credits could be given as a reward to enhance reporting of suspected serious adverse reactions. In the UK, for example, practitioners who promptly report any serious unexpected suspected adverse reactions to the MHRA could receive credits through the Quality Outcomes Framework system, although this could lead to over-reporting of suspected adverse drug reactions. In addition, more rigorous monitoring and verification of deaths and reasons for dropping out during clinical trials is warranted [15].

If an agency receives a report of a death associated with a medication, it could suspend the product temporarily while awaiting further information, and contact other agencies for information about other reports. Systematic reviews of such reports could then be carried out and would be enabled by the introduction of international standards for reporting suspected adverse reactions [55,56]. Withdrawal of a product would not necessarily be warranted on the basis of a single report, but several reports from disparate sources would arouse suspicion of a testable association. Natalizumab was withdrawn temporarily after deaths had been reported, and reintroduced later, with safeguards, because the benefit-to-harm balance was perceived to be favourable [57].

Finally, the criteria for determining that an event is a signal could have a lower threshold when death is the event. For example, in analysing deaths in comparative trials (e.g., using placebo), the 90% confidence interval could be used as a less strict criterion than usual for deciding whether death was a signal worthy of further investigation.

## Conclusions

We have identified 95 products for which death was cited as a reason for withdrawal. The interval between launch date and reports of deaths has shortened over the past few decades, and this could be because of better reporting of suspected adverse reactions or stricter regulation. However, many withdrawals still occur more than 1 or 2 years after the reports of deaths begin to appear. Furthermore, there are discrepancies in the patterns of drug withdrawals in different countries, with greater delays in Africa and Asia. These delays and discrepancies could be mitigated by encouraging prescribers and investigators to report serious suspected adverse reactions, by swifter regulatory action when reports appear, and by international co-ordination of reports.

## Additional files

Additional file 1:
**Medline search strategy for identification of report dates for first report of death and first date of withdrawal.**
Additional file 2: Table S1. Medicinal products withdrawn because of drug-attributed deaths.

## Abbreviations

FDA: US Food and Drug Administration
MAH: Marketing authorization holder
MHRA: UK Medicines and Healthcare products Regulatory Agency
WHO: World Health Organization
