# Supplementary material for: Delays in the post-marketing withdrawal of drugs to which deaths have been attributed: a systematic investigation and analysis
Source: BMC Med. 2015 Feb 5;13:26. doi: 10.1186/s12916-014-0262-7 (PMC4318389; doi:10.1186/s12916-014-0262-7)
Supplement: Additional file 1: — Medline search strategy for identification of report dates for first report of death and first date of withdrawal. [file 12916_2014_262_MOESM1_ESM.pdf]

Appendix 1: Medline search strategy for identification of report dates for first report of death, first date of withdrawal

“[Drug name]” AND “fatal*”

“[Drug name]” AND “fatality”

“[Drug name]” AND “death”

“[Drug name]” AND “toxicity”

“[Drug name]” AND “adverse”

“[Drug name]” AND “withdrawal”

“[Drug name]” AND “withdrawn”

“[Drug name]” AND “recall”

“[Drug name]” AND “voluntary recall”

“[Drug name]” AND “banned”

“[Drug name]” AND “prohibited”

[Sort by Publication date]
