# Supplementary material for: Delays in the post-marketing withdrawal of drugs to which deaths have been attributed: a systematic investigation and analysis
Source: BMC Med. 2015 Feb 5;13:26. doi: 10.1186/s12916-014-0262-7 (PMC4318389; doi:10.1186/s12916-014-0262-7)
Supplement: Additional file 2: Table S1. — Medicinal products withdrawn because of drug-attributed deaths. [file 12916_2014_262_MOESM2_ESM.pdf]

Table 1: Medicinal products withdrawn because of drug-attributed deaths

Medicinal product	Launch year	First death reported	Year first withdrawn*	Countries of withdrawal[†‡]	Death attributed to	Level of evidence[§]
Alosetron	2000	2000	2000	USA; re-introduced with restrictions in 2002	Ischaemic colitis	4
Alpidem	1991	1994	1994	France	Hepatotoxicity	4
Aminophenazone	1887	1936	1965	10 European, 14 Asian, & 4 African countries; Australia; USA; Chile; Brazil; Venezuela	Agranulocytosis	4
Amobarbital[¶]	1961	1979	1985	Sweden	Respiratory depression	4
Aprobarbital[¶]	1920	1966	1985	Sweden	Respiratory depression	4
Azaribine	1975	1976	1976	USA; Thailand; Mauritius; Saudi Arabia; Venezuela	Thromboembolism	4
Beclobrate	1985	1990	1990	Switzerland	Hepatotoxicity	4
Benoxaprofen	1980	1982	1982	Worldwide	GI haemorrhage	4
Benzarone	1964	1992	1992	Germany	Hepatotoxicity	4
Bicalutamide (150 mg)	1995	2001	2003	Canada, UK	"Accelerated deaths"	2
Bismuth salts	1875	1930	1978	France; Egypt; Japan; Greece; Austria; Bangladesh	Cardiotoxicity, neurotoxicity	4
Boric acid and borates	1875	1945	1981	Ireland; Malaysia	Neurotoxicity	4
Bromocriptine mesylate	1976	1995	1994	USA	Cardiotoxicity, neurotoxicity	4
Bromfenac Na	1997	1998	1998	USA; Saudi Arabia	Hepatotoxicity	4
Buformin	1957	1976	1978	Germany; Belgium; Ireland; Austria	Metabolic acidosis	4
Bunamiodyl	1958	1962	1964	USA, Sweden	Nephrotoxicity	4
Buprenorphine[¶]	1978	1991	1986	Egypt	Respiratory depression	4
Celecoxib	1999	2006	2011	European Union	Cardiotoxicity	1
Cerivastatin	1990	1998	2001	Worldwide	Rhabdomyolysis; renal failure	4
Cianidanol	1976	1985	1988	Worldwide	Haemolytic anemia	4
Cisapride monohydrate	1993	1993	2000	USA; UK; Canada; Philippines; Mauritius; Brunei; Armenia	Cardiotoxicity	4
Clometacin	1977	1981	1987	France	Hepatotoxicity	4
Clozapine	1972	1975	1975	USA; Finland	Agranulocytosis	4
Coumarin (synthetic)	1996	1996	1996	France; Australia	Hepatotoxicity	4
Dextropropoxyphene +	1978	1987	2005	UK	Hepatotoxicity	4

paracetamol (co-proxamol) ¶						
Dibenzepin hydrochloride¶	1968	1973	1983	Sweden	Cardiotoxicity	4
Diiododiethyltin & isolinoleic ester	1954	1955	1957	France	Cerebral edema	4
Dithiazanine iodide	1959	1960	1964	USA; France; Chad; Italy; Cuba	Metabolic acidosis	4
Domperidone (Inj)	1979	1982	1985	Worldwide	Cardiotoxicity	4
Doxycycline (IV)	1960	1984	1989	France	Anaphylaxis	4
Ebrotidine	1997	1997	1998	Spain; Peru	Hepatotoxicity	4
Encainide	1980	1991	1991	UK	Cardiotoxicity	4
Ethyl nitrite	1850	1977	1980	USA	Methaemoglobinemia	4
Felbamate	1993	1993	1994	USA, European Union	Hepatotoxicity; aplastic anaemia	2&4
Fenclofenac	1978	1984	1985	Worldwide	Toxic epidermal necrolysis	4
Fenoterol	1971	1987	1990	New Zealand	Increased mortality due to asthma	3
Fipexide	1973	1992	1991	France	Hepatotoxicity	4
Flosequinan	1992	1992	1993	UK	Cardiotoxicity	2
Grepafloxacin	1997	1999	1999	Worldwide	Cardiotoxicity	2&4
Heptabarb¶	1956	1984	1984	Sweden	Respiratory depression	4
Hexobarbital¶	1945	1962	1984	Sweden	Respiratory depression	4
Indoprofen	1976	1982	1983	Worldwide	Gastrointestinal hemorrhage	4
Isaxonine phosphate	1981	1983	1984	France	Hepatotoxicity	4
Isoxicam	1983	1985	1985	Worldwide	Fatal skin reactions	4
Ketorolac injection	1990	1993	1993	Germany; France	Gastrointestinal hemorrhage	4
Loperamide (syrups & drops)	1975	1989	1990	Worldwide	Paralytic ileus	4
L-tryptophan	1963	1989	1989	Worldwide	Eosinophilia-myalgia syndrome (EMS)	3
Metamizole	1921	1952	1974	7 European and 11 Asian countries; Australia; USA; Ghana; Zimbabwe; Morocco	Agranulocytosis	4
Mibefradil	1997	1998	1998	USA; UK; Peru; South Africa; Jamaica; Bulgaria; Armenia	Drug interactions	4
Muzolimine	1985	1987	1987	Worldwide	Nephrotoxicity	4
Naftidrofuryl oxalate IV	1984	1994	1995	France; Germany; restricted in the UK	Anaphylaxis	4
Nebacumab	1991	1993	1993	Worldwide	“Accelerated deaths”	2

Nefazodone	1994	2002	2003	European Union; Canada; Singapore	Hepatotoxicity	4
Nimesulide	1986	2000	2002	Ireland; Israel; Bangladesh; Spain	Hepatotoxicity	4
Nitrefazole	1980	1984	1984	Germany; Austria	Hepatotoxicity	4
Nomifensine	1976	1985	1986	Worldwide	Haemolytic anaemia	4
Orgotein	1968	1990	1990	Switzerland; Germany; Portugal	Anaphylaxis	4
Osmosin	1975	1983	1983	UK	Adherence to intestinal wall	4
Oxomemazine	1937	1979	1991	European Union; USA	Respiratiory depression in children (SIDS)	4
Oxyphenbutazone	1955	1974	1984	Worldwide	Bone marrow suppression	4
Oxyphenisatine acetate	1955	1972	1970	Worldwide	Hepatotoxicity	4
Pemoline	1975	1989	2005	Worldwide	Hepatotoxicity	4
Pentobarbital[¶]	1930	1953	1985	Sweden	Respiratory depression	4
Phenformin HCl	1957	1963	1970	14 European & 3 Asian countries; Canada; New Zealand; Brazil; USA; Ethiopia	Metabolic acidosis	4
Phenobarbital[¶]	1912	1929	1985	Sweden	Respiratory depression	4
Phenylbutazone	1949	1953	1984	6 European, 6 Asian & 3 African countries; Chile	Bone marrow suppression	4
Pirprofen	1982	1986	1990	Worldwide	Hepatotoxicity	4
Practolol	1970	1977	1994	Germany; Norway; Venezuela	Hypersensitivity	4
Prenylamine	1960	1988	1989	Worldwide	Cardiotoxicity	4
Pumactant	1992	2000	2000	UK	"Accelerated deaths"	2
Rapacuronium bromide	1999	2000	2001	USA	Bronchospasm	4
Rofecoxib	1999	2002	2004	Worldwide	Cardiotoxicity	1
Rosiglitazone	1999	2007	2011	UK; New Zealand; South Africa	Cardiotoxicity	1,2,3
Sertindole	1996	1996	1998	UK, Bulgaria	Cardiotoxicity	2
Sibutramine	2001	2002	2010	European Union; 4 Asian countries; Australia; Canada; Mexico; New Zealand; USA	Cardiotoxicity	4
Sitaxentan sodium	2006	2002	2010	Worldwide	Hepatotoxicity	4
Somatropin (pituitary-derived)	1973	1985	1985	Cyprus; Ireland; UK; Egypt; Turkey; New Zealand; Netherlands; USA; Oman	Creutzfeldt-Jakob disease	4
Sorivudine	1993	1993	1993	Germany; Japan	Interaction with 5-fluorouracil	4
Suloctidil	1975	1983	1985	Worldwide	Hepatotoxicity	4

Technetium (99mTc) fanolesomab	2004	2005	2005	USA	Cardiotoxicity	4
Temafloxacin	1991	1992	1992	Worldwide	Haemolytic anaemia	4
Temazepam (gel capsules) ¶	1969	1992	2004	Australia	Respiratory depression	3
Terfenadine	1985	1991	1997	3 European, 2 African, 2 Asian & 3 South American countries; USA	Cardiotoxicity	4
Terodliline	1965	1989	1991	Worldwide	Cardiotoxicity	4
Thenalidine	1953	1958	1958	USA; UK; Sweden; France; Cyprus; Australia; Finland; Norway	Neutropenia	4
Tienilic acid	1976	1980	1980	Greece; Philippines; USA; Brazil; Germany; Panama; France; India; Venezuela	Hepatotoxicity	4
Tolcapone	1998	1998	1998	UK; Australia; Ireland; Spain; Portugal; Lithuania; Bulgaria	Hepatotoxicity	4
Tolrestat	1982	1995	1996	Worldwide	Hepatotoxicity	2
Torcetrapib	1999	2006	2006	Worldwide	Cardiotoxicity	2
Troglitazone	1997	1997	1997	UK; Switzerland; Peru	Hepatotoxicity	4
Trovafloxacin	1997	1996	1999	European Union; Philippines; Syria	Hepatotoxicity	2&4
Vinbarbital¶	1939	1975	1984	Sweden	Respiratory depression	4
Vitamin E (IV)	1983	1984	1984	USA	Hepatotoxicity	4
Zipeprol HCl¶	1982	1991	1993	Brazil; Philippines	Respiratory depression	4
Zomepirac Na	1979	1983	1983	Worldwide	Anaphylaxis	4

*For any reason; some drugs have been withdrawn for one or more indications but not for others
†The European Union refers to the European Medicines Agency member states; European, African, Asian, or South American countries refers to nations geographically located in these regions
‡Worldwide = in all countries in which it was marketed
¶Drugs withdrawn because of fatal intoxication
§Based on the Oxford Centre for Evidence-Based Medicine Levels of Evidence. Level 1: Systematic review of randomized trials, systematic review of nested case-control studies, Level 2: Individual randomized trial or (exceptionally) observational study with dramatic effect; Level 3: Non-randomized controlled cohort/follow-up study (post-marketing surveillance); Level 4: Case-series, case-control, or historically controlled studies; Level 5: Mechanism-based reasoning
